# Supplementary material for: Critical slowing down as a biomarker for seizure susceptibility
Source: Nat Commun. 2020 May 1;11:2172. doi: 10.1038/s41467-020-15908-3 (PMC7195436; doi:10.1038/s41467-020-15908-3)
Supplement: Supplementary file 1 — Supplementary information [file 41467_2020_15908_MOESM1_ESM.pdf]

Critical slowing down as a biomarker for seizure susceptibility

Maturana et. al. 2020: Supplementary information

Supplementary information

Supplementary Note 1

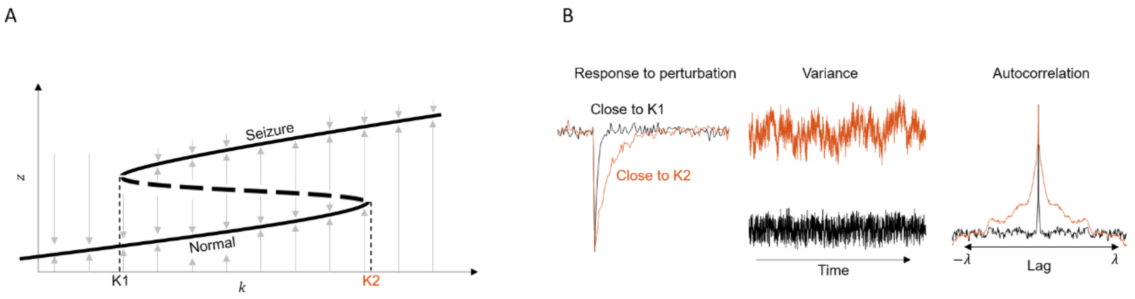

Supplementary Figure 1: Conceptualization of critical slowing. A) A bifurcation diagram showing the fixed points of a dynamical system (black lines) for different values of k. The distance between the normal and unstable fixed point (dashed line) can be thought of as the perturbation size necessary to transition into the seizure state. B) As k is driven towards K2, the system exhibits critical slowing, which is characterized by an increase in the response time constant. This results in an increase response time to small perturbations, increase in signal variance and autocorrelation.

Detailed description of critical slowing

Supplementary Figure A describes a typical fold bifurcation, where the state z can assume multiple solutions. A general equation governing the dynamics of z is

$$\frac{dz}{dt} = F(z, k). \quad (1)$$

The system can be linearized about a point K and solved for z to describe the rate of exponential decay to small perturbations. Assuming that $F(z, k)$ is a normal operator, the solution has a form given by

$$z(t) = Z_K + Ce^{at}\ . \quad (2)$$

Here, Z_K represents the fixed point of z at k=K, and C represents the perturbation size. The variable a depends on the point at which Equation (2) is linearized. Assuming linearization occurs about points k_0, r_0 and Z_k, then $a = 3Z_k^2 + r_0$. The system time constant is given by $\tau = \frac{1}{a}$. For stable states, the exponent, a, is negative (i.e. the exponential decays) and for unstable states, the exponent is positive (i.e. increasing exponential). Under these one-dimensional assumptions, if z is perturbed from the normal state, it will settle down to its steady state with a time constant dictated by a. Critical slowing down is a phenomenon that emerges as z is driven towards K2. Critical slowing down arises from the fact that driving k towards K2 results in a becoming larger. In other words, the time constant

of the system diverges, leading to an increased recovery time to perturbations, increased signal variance and autocorrelation (Supplementary Figure 1B). The change in the system time constant can be measured by analyzing the autocorrelation of the signal.

Relationship between autocorrelation function and signal time constant

Building from Equation (2), we can derive the relationship between the linearized system response and the autocorrelation signal. The autocorrelation function results in a function with the same time constant as the linearized response function. The proof is shown below:

The autocorrelation for an ergodic signal is given by $R(\lambda) = \frac{1}{T}\int_0^T f(t)f(t+\lambda)dt$.

Letting $f(t) = Z_K + Ce^{at}$.

$$R(\lambda) = \frac{1}{T}\int_0^T (Z_K + Ce^{at})\left(Z_K + Ce^{a(t+\lambda)}\right)dt$$

$$R(\lambda) = \frac{1}{T}\int_0^T Z_K^2 + Z_K C(e^{at} + e^{a(t+\lambda)}) + C^2 e^{at+a(t+\lambda)}dt$$

$$R(\lambda) = \frac{1}{T}\int_0^T Z_K^2 + Z_K Ce^{at} + Z_K Ce^{a\lambda}e^{at} + C^2 e^{a\lambda}e^{2at}\, dt$$

$$R(\lambda) = \frac{1}{T}\left[Z_K^2 t + \frac{Z_K C}{a}e^{at} + \frac{Z_K Ce^{a\lambda}}{a}\, e^{at} + \frac{C^2 e^{a\lambda}}{2a}e^{2at}\right]_0^T$$

$$R(\lambda) = \frac{1}{T}(Z_K^2 T + \frac{Z_K C}{a}e^{aT} + \frac{Z_K Ce^{a\lambda}}{a}\, e^{aT} + \frac{C^2 e^{a\lambda}}{2a}e^{2aT} - \frac{Z_K C}{a} - \frac{Z_K Ce^{a\lambda}}{a} - \frac{C^2 e^{a\lambda}}{2a})$$

$$R(\lambda) = \frac{1}{T}\left(Z_K^2 \mathrm{T} + \frac{Z_K C}{a}e^{aT} - \frac{Z_K C}{a}\right) + \frac{1}{\mathrm{T}}e^{a\lambda}\left(\frac{Z_K Ce^{aT}}{a} + \frac{C^2 e^{2aT}}{2a} - \frac{Z_K C}{a} - \frac{C^2}{2a}\right)$$

Letting $A = \frac{1}{T}\left(Z_K^2 \mathrm{T} + \frac{Z_K C}{a}e^{aT} - \frac{Z_K C}{a}\right)$ and $B = \frac{1}{T}\left(\frac{Z_K Ce^{aT}}{a} + \frac{C^2 e^{2aT}}{2a} - \frac{Z_K C}{a} - \frac{C^2}{2a}\right)$ we get

$$R(\lambda) = A + Be^{a\lambda},$$

which has the same form as Equation (2) and the same time constant $\tau = \frac{1}{a}$. Note that the autocorrelation at zero, $R(0)$, is equivalent to the signal energy. As a proxy to measuring the time constant, the width at half maximum of the autocorrelation function can be used. In this case, the width will increase monotonically with increasing time constant.

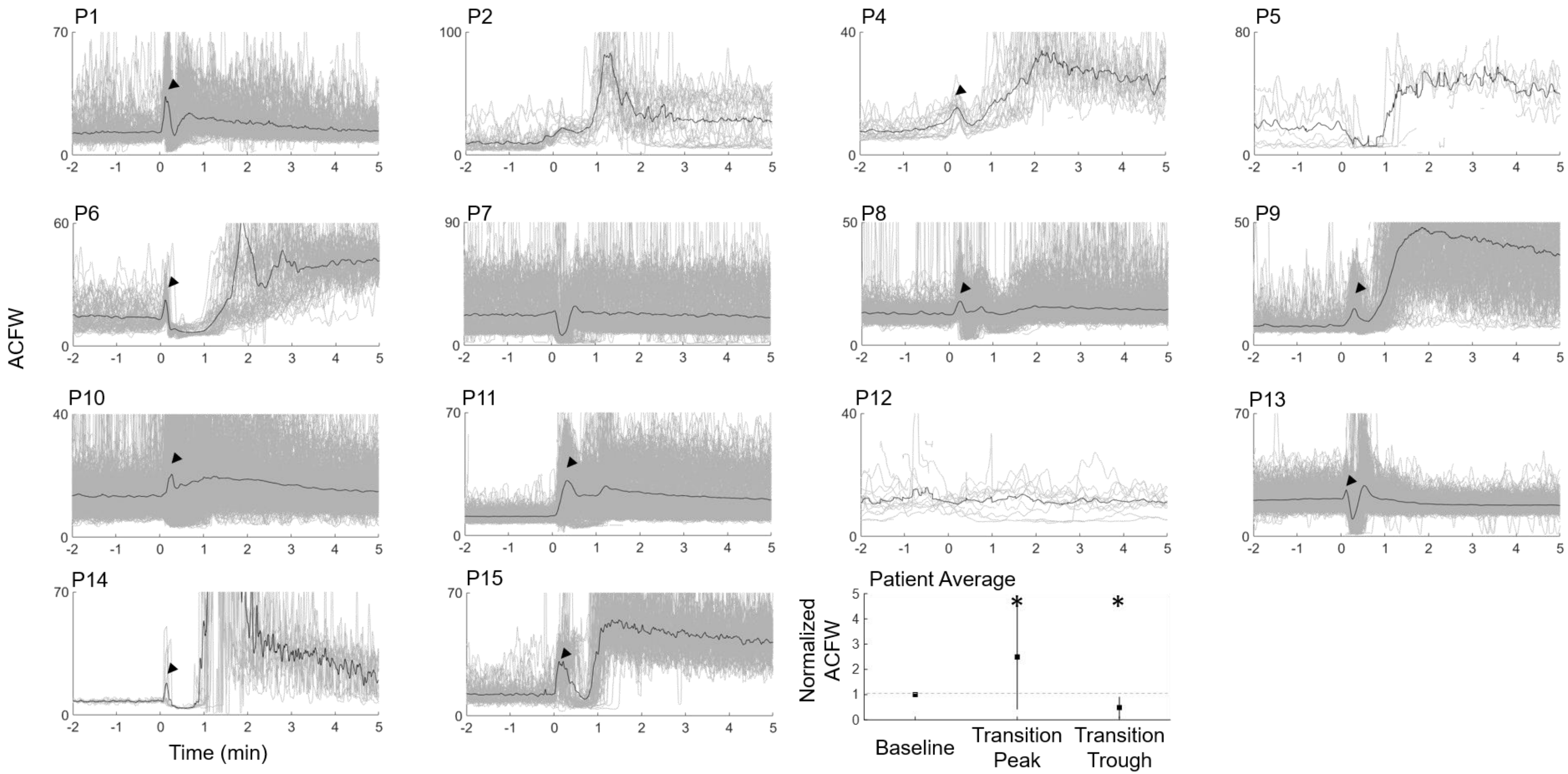

Supplementary Figure 2: The autocorrelation width (ACFW) is shown for all seizures (gray lines) across the 14 patients. A sharp drop in the ACFW close to the onset time is a hallmark of a critical transition. This hallmark is visible for all seizures except in Patient 12. Many patients showed a rapid approach to the critical point, as defined by a sharp increase in ACFW prior to the drop (black arrows). The black lines show the average across all seizures. Time zero defines the clinically defined seizure onset time. The Patient average compares a baseline period 5 minutes prior to the seizure, the peak ACFW and the subsequent trough for all seizures across all patients (excluding Patient 12). Squares denote the mean and lines show one standard deviation. Stars denote values that were significantly different from baseline.

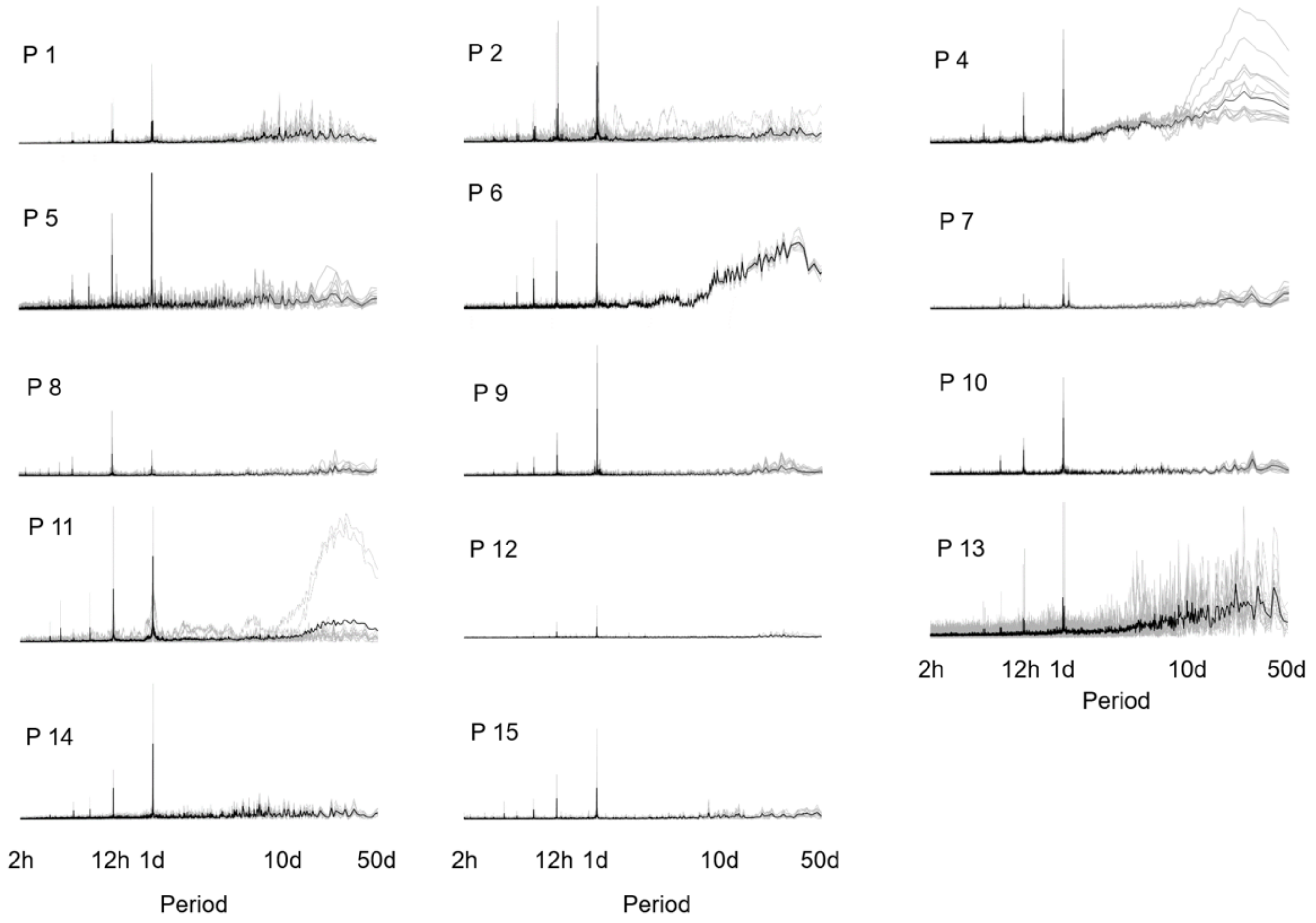

Supplementary Figure 3: Fourier transform (FT) of the raw autocorrelation signal for each patient. The transform shows peaks at 12 and 24 hours for every patient, demonstrating a strong daily rhythm in the signal. Some patients also had peaks at higher periods (e.g. Patients 4 and 6). In each subplot, the gray lines represent the FT for each of the sixteen channels, and the black represents the mean across channels.

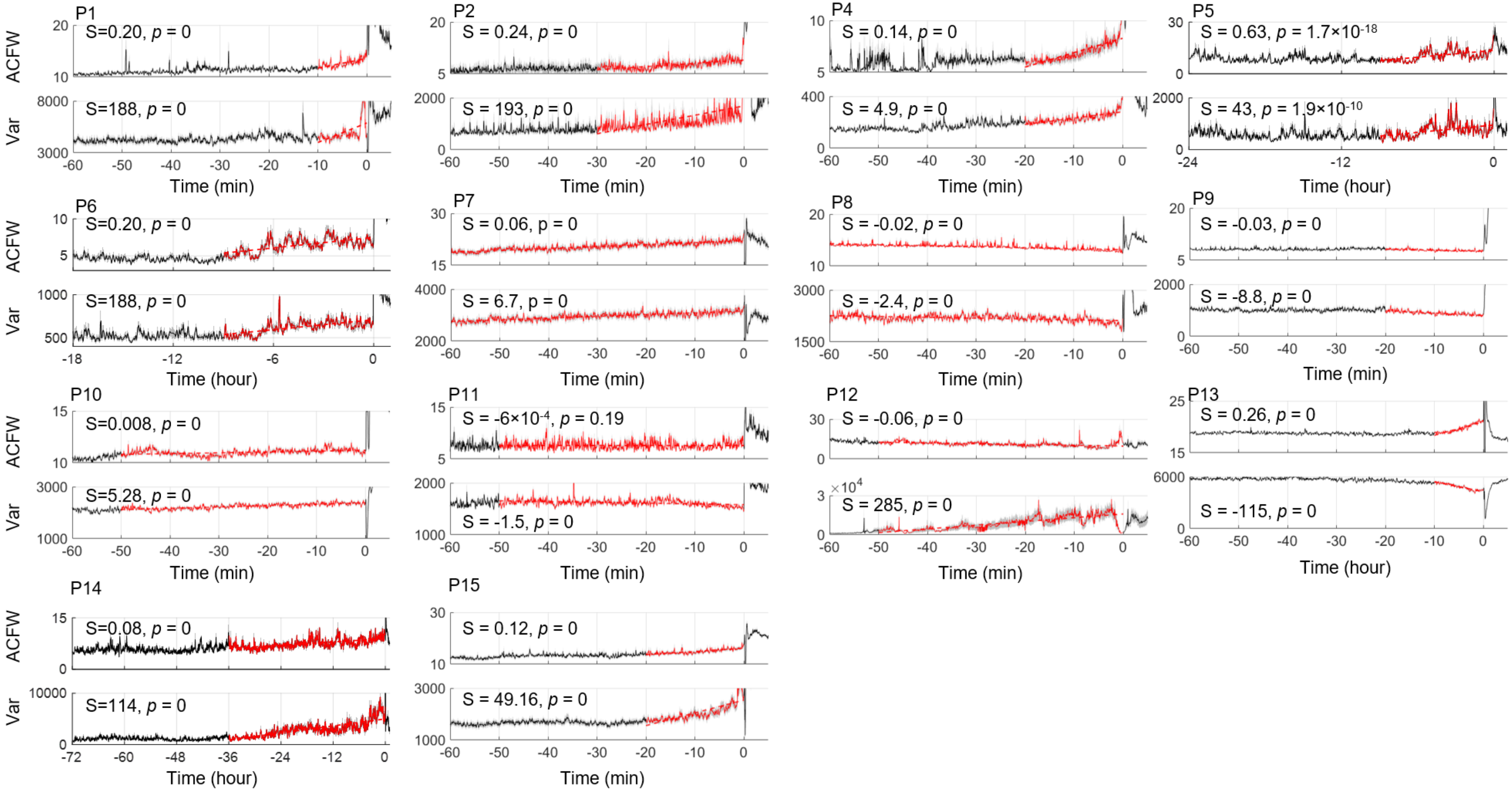

Supplementary Figure 4: The autocorrelation width (ACFW) and variance (Var) is shown for lead seizures across the 14 patients. The mean across all lead seizures (black) and standard error (gray) is shown, along with the region to which a linear regression was fitted (red). S refers to the signal slope (change in signal per unit time) of the linear regression. The *p* value refers to the slope being significantly different to zero. Most regressions slopes were significant. For example, the slope was significantly smaller than zero (negative) in patient 9, while the ACFW slope was not significant (i.e. flat) in patient 11. Time zero defines the clinically defined seizure onset time. Note the difference in time scale for each patient.

Supplementary Note 2

Dynamical system example

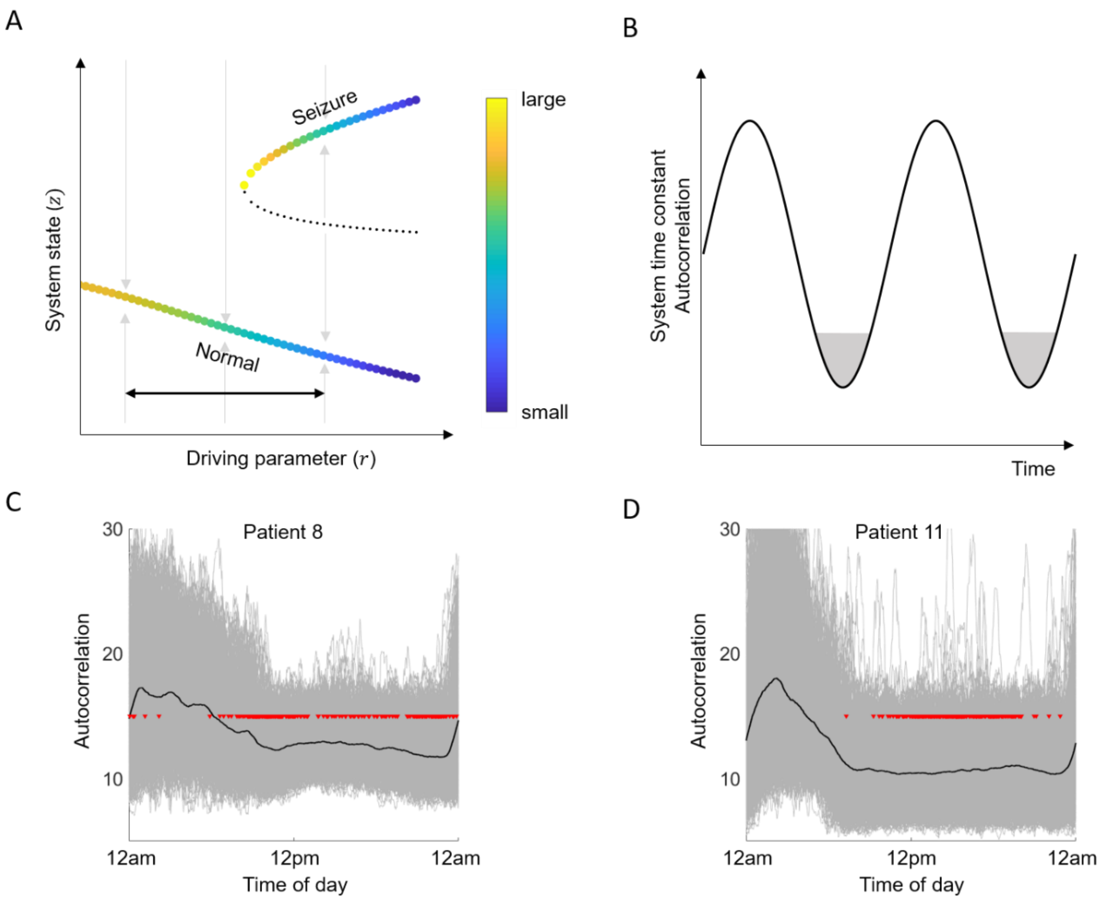

Supplementary Figure 5: Example dynamical system where seizures occur during periods of low autocorrelation. A) The system fixed points are given for different values of r. The color represents the linearized system's time constant. As r is varied in the region given by the black arrow, the system transitions from having one stable fixed point, to two stable fixed points separated by an unstable fixed point. B) As r is varied over time, the autocorrelation changes such that seizures are only possible during low values of the autocorrelation (shaded region). C-D) The autocorrelation across hour of day is given for Patient 8 (C) and Patient 11 (D). Gray lines represent raw autocorrelation values and the black lines represent the average across all days. The red triangles represent seizures.

Throughout the text, we use an example dynamical system given by

$$\frac{dz}{dt} = -z^3 + (1 \times 10^{-3})rz + (1 \times 10^{-3})k. \quad (3)$$

Fixing $r > 0$ and solving for z while varying k produces the fixed points in Figures 1 and Supplementary Figure 1. However, if we instead fix $k<0$ and vary r, we obtain fixed points such as those shown in Supplementary Figure 5A. If r is varied in the region shown by the black arrow, we see that the system changes from mono-stable for small values of r, to bi-stable for larger values. For this system, the seizure state becomes possible only for large values of r, which corresponds to low

values of the time constant (Supplementary Figure 5B, shaded region). Two example autocorrelation curves relative to time of day is shown for Patients 8 and 11 (Supplementary Figure 5C,D). Seizures occurred only at the trough of the autocorrelation signal, similar to the example shown in Supplementary Figure 5B. In these examples, the autocorrelation is modulated on slow (daily) time scales. The pathway to seizure could be perturbation mediated or could results from a fast change in k such that the critical point leading to seizures is approached (i.e. Figure 1A).

Patient 1

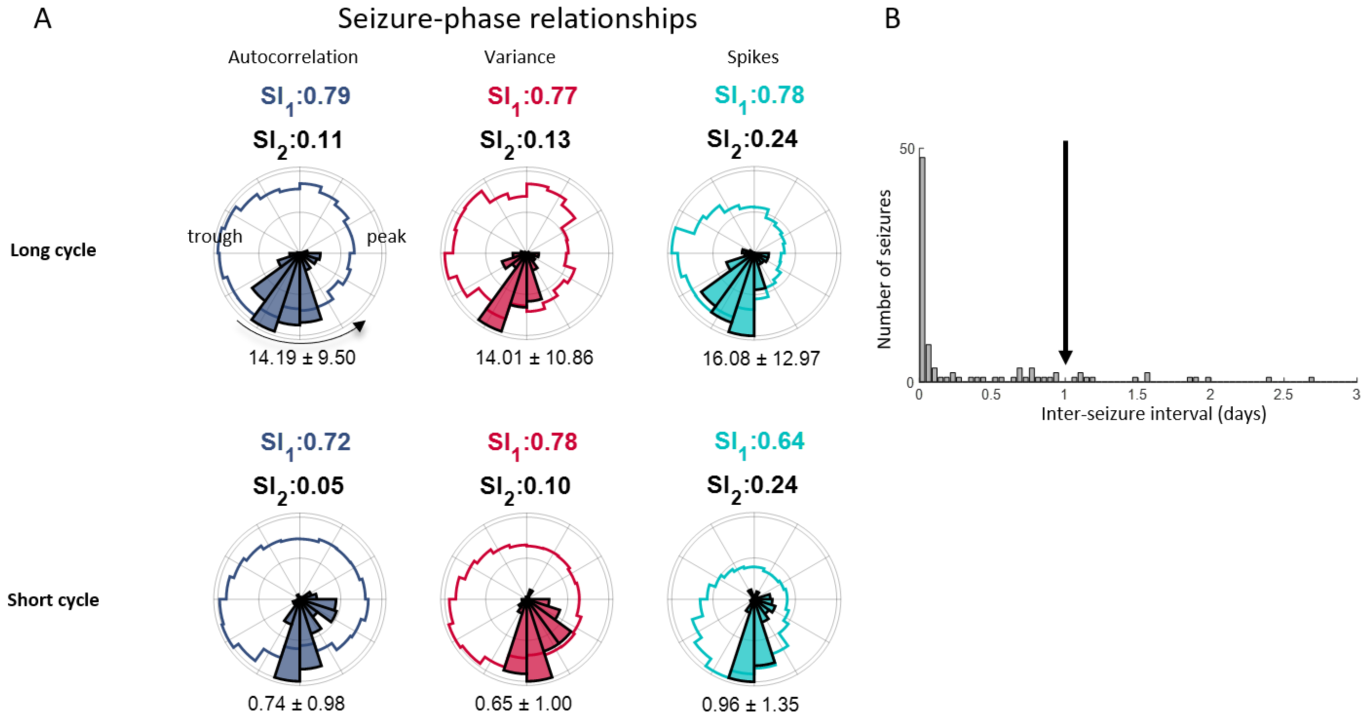

Supplementary Figure 6: Results summary for Patient 1. A) The Synchronization Indices (SI_1) for the autocorrelation (gray-blue), variance (red) and spikes (cyan) quantify the synchrony between seizures and the underlying signals. A high SI_1 corresponds to good alignment between seizures and the phase of the signal. The SI for the signals (SI_2) quantifies the phase uniformity of the signal. A low SI_2 demonstrates that the signal is periodic and that all phases are equally represented. The numbers below each subplot denote the average cycle duration (mean and standard deviation). B) The inter-seizure interval showed that a very high proportion of seizures occurred within 1 hour of a previous seizure. We set the lead seizure cut-off to 1 day.

Patient 2

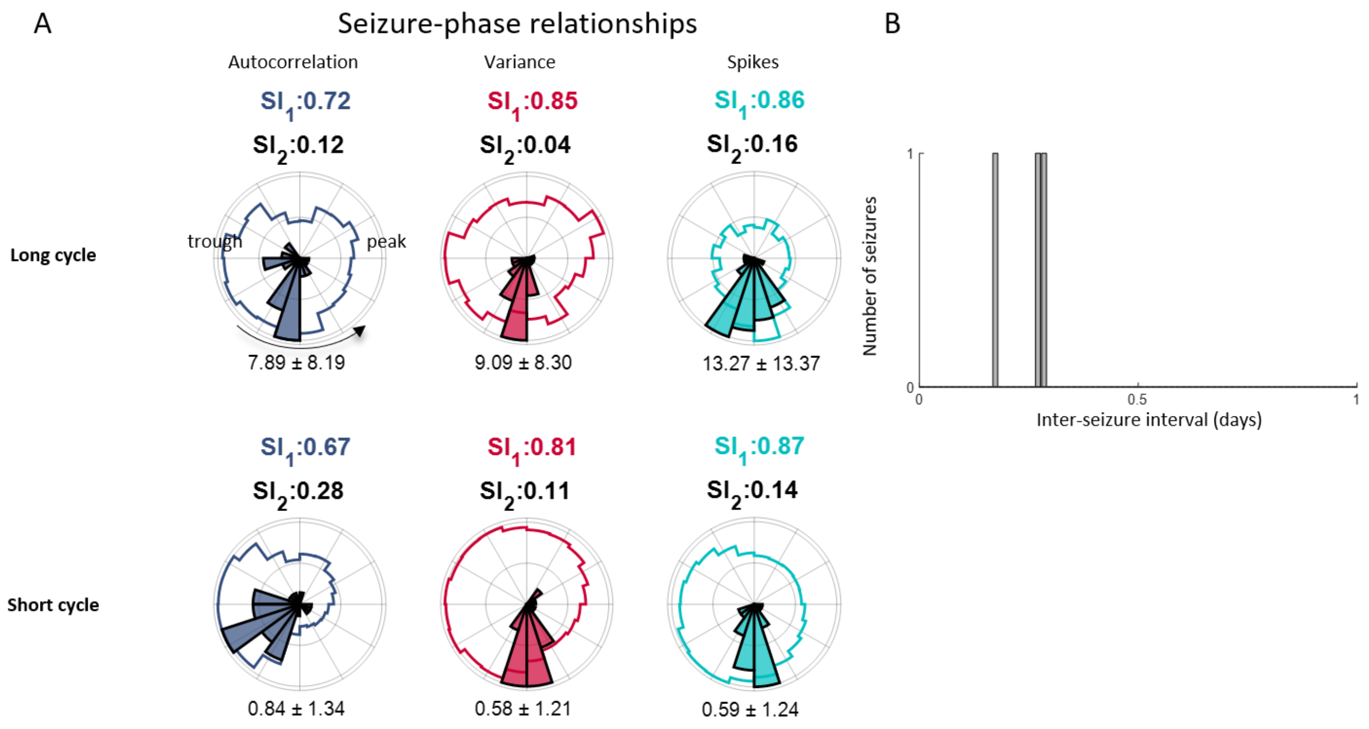

Supplementary Figure 7: Results summary for Patient 2. A) The Synchronization Indices (SI_1) for the autocorrelation (gray-blue), variance (red) and spikes (cyan) quantify the synchrony between seizures and the underlying signals. A high SI_1 corresponds to good alignment between seizures and the phase of the signal. The SI for the signals (SI_2) quantifies the phase uniformity of the signal. A low SI_2 demonstrates that the signal is periodic and that all phases are equally represented. The numbers below each subplot denote the average cycle duration (mean and standard deviation). B) The inter-seizure interval. This patient had too few seizures to meet the seizure clustering criteria.

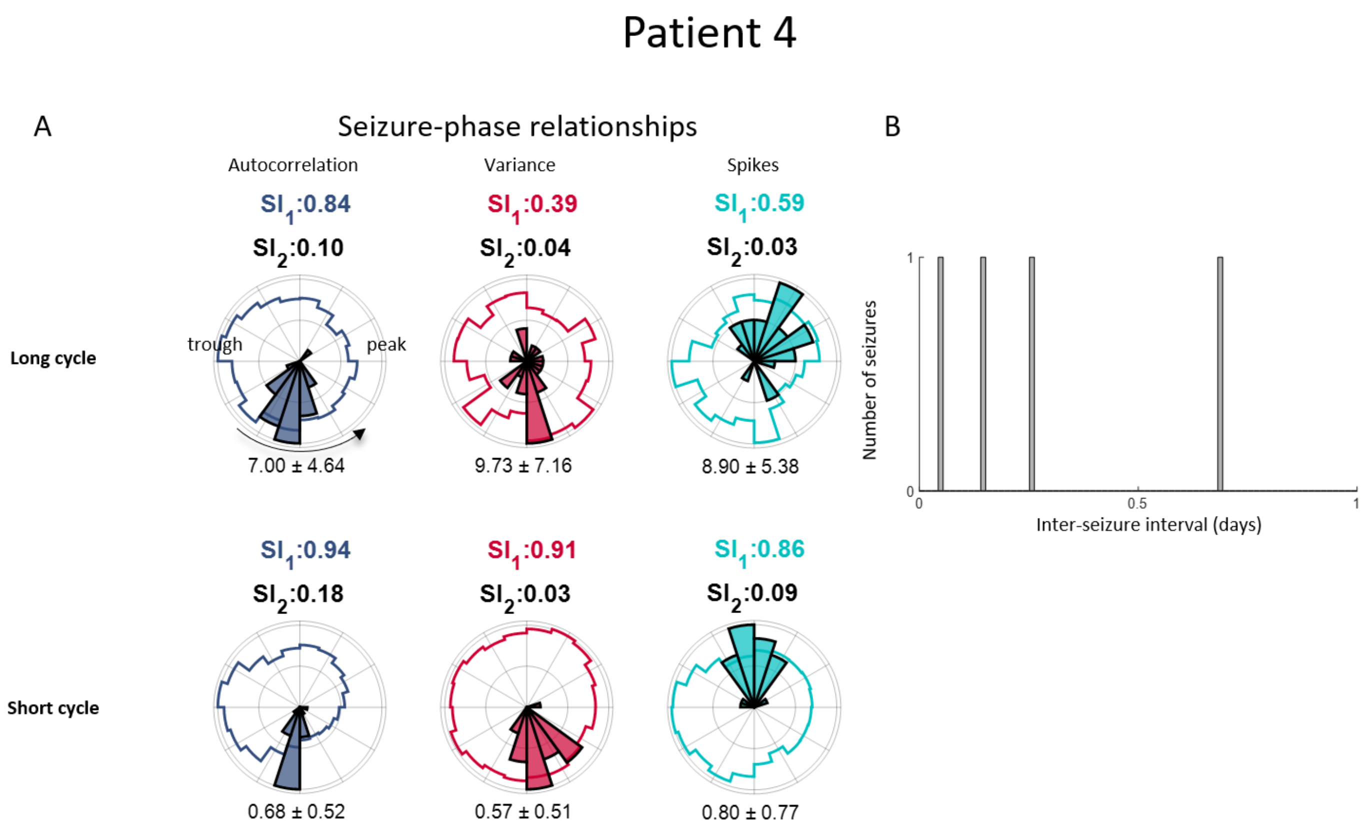

Supplementary Figure 8: Results summary for Patient 4. A) The Synchronization Indices (SI_1) for the autocorrelation (gray-blue), variance (red) and spikes (cyan) quantify the synchrony between seizures and the underlying signals. A high SI_1 corresponds to good alignment between seizures and the phase of the signal. The SI for the signals (SI_2) quantifies the phase uniformity of the signal. A low SI_2 demonstrates that the signal is periodic and that all phases are equally represented. The numbers below each subplot denote the average cycle duration (mean and standard deviation). B) The inter-seizure interval. This patient had too few seizures to meet the seizure clustering criteria.

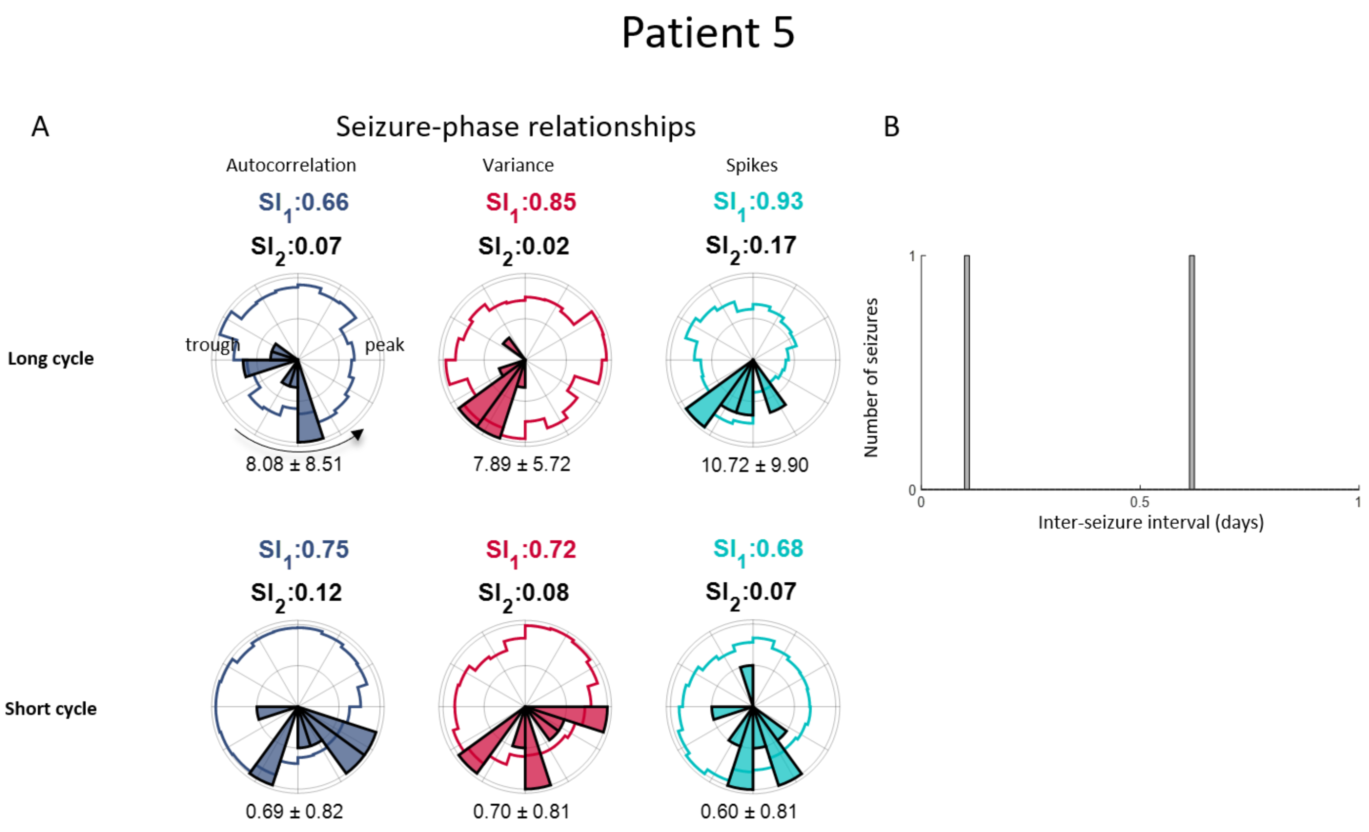

Supplementary Figure 9: Results summary for Patient 5. A) The Synchronization Indices (SI_1) for the autocorrelation (gray-blue), variance (red) and spikes (cyan) quantify the synchrony between seizures and the underlying signals. A high SI_1 corresponds to good alignment between seizures and the phase of the signal. The SI for the signals (SI_2) quantifies the phase uniformity of the signal. A low SI_2 demonstrates that the signal is periodic and that all phases are equally represented. The numbers below each subplot denote the average cycle duration (mean and standard deviation). B) The inter-seizure interval. This patient had too few seizures to meet the seizure clustering criteria.

Patient 6

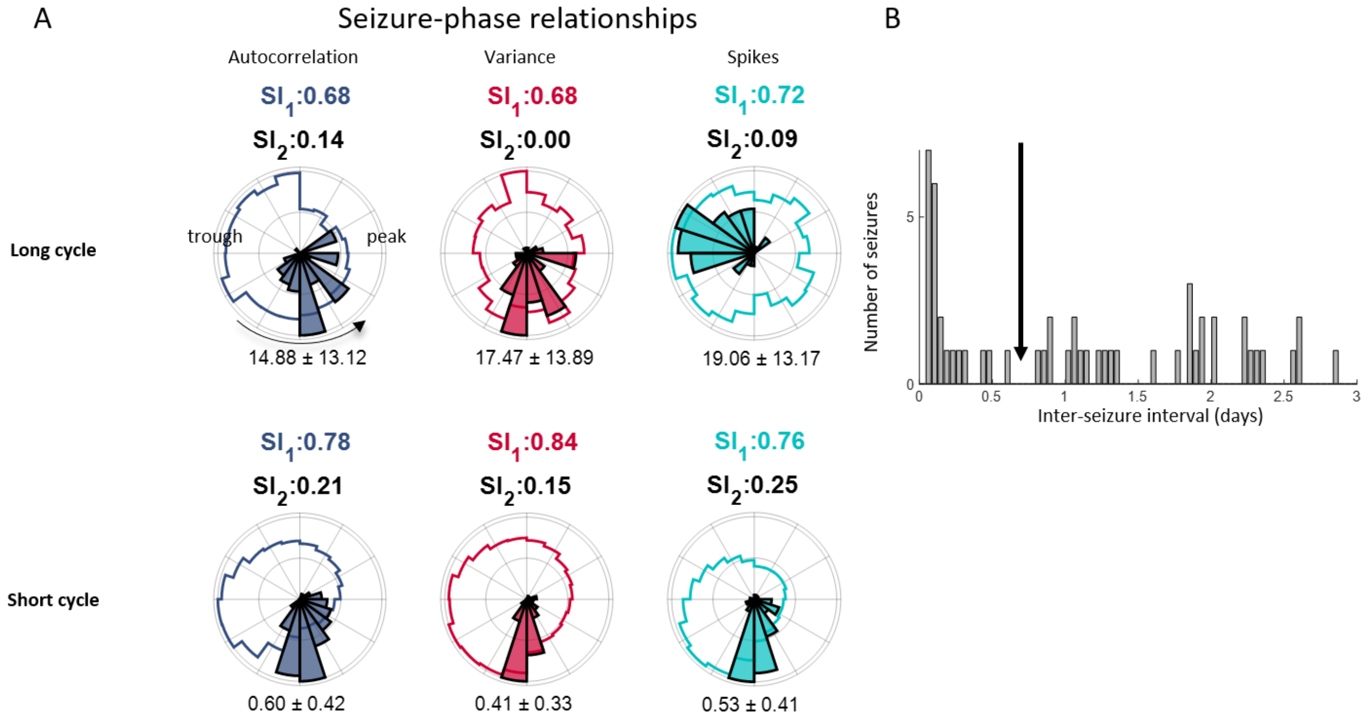

Supplementary Figure 10: Results summary for Patient 6. A) The Synchronization Indices (SI_1) for the autocorrelation (gray-blue), variance (red) and spikes (cyan) quantify the synchrony between seizures and the underlying signals. A high SI_1 corresponds to good alignment between seizures and the phase of the signal. The SI for the signals (SI_2) quantifies the phase uniformity of the signal. A low SI_2 demonstrates that the signal is periodic and that all phases are equally represented. The numbers below each subplot denote the average cycle duration (mean and standard deviation). B) The inter-seizure interval. This patient had peaks at approximately 1 and 2 days. We set the cut-off at 0.7 days (17 hours).

Patient 7

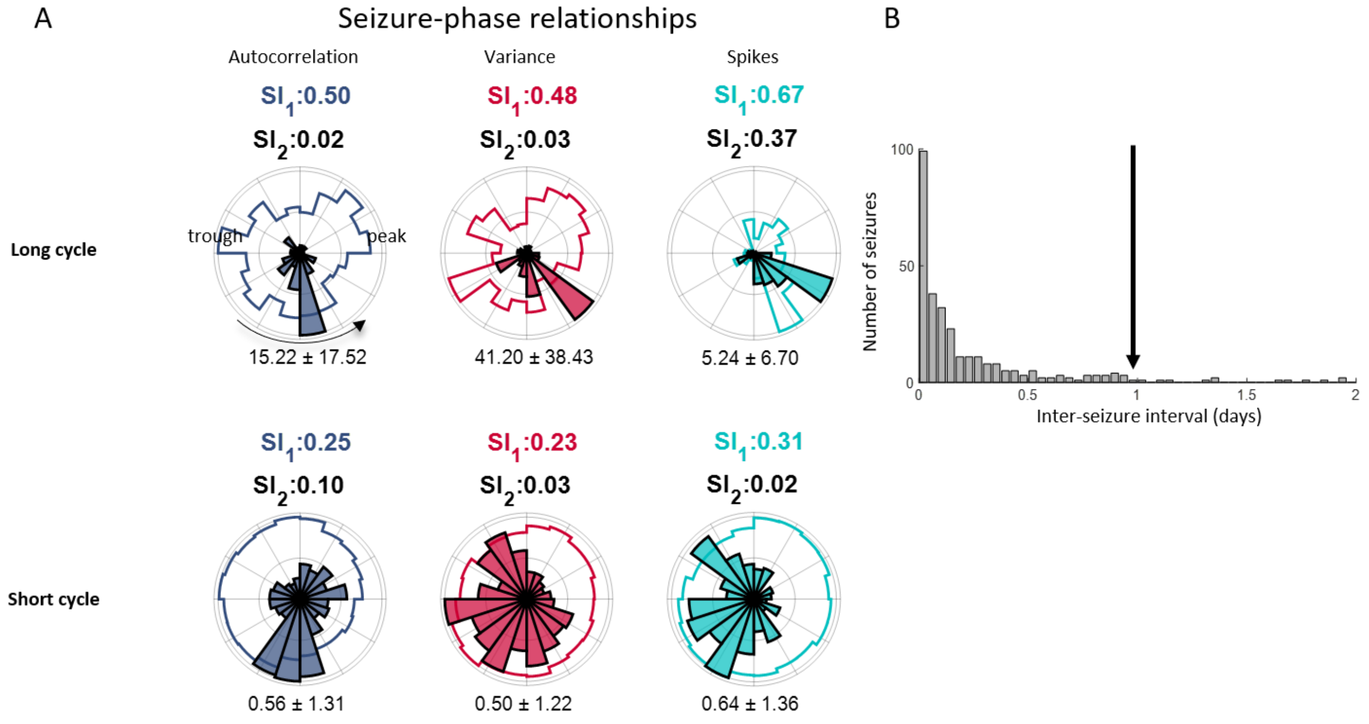

Supplementary Figure 11: Results summary for Patient 7. A) The Synchronization Indices (SI_1) for the autocorrelation (gray-blue), variance (red) and spikes (cyan) quantify the synchrony between seizures and the underlying signals. A high SI_1 corresponds to good alignment between seizures and the phase of the signal. The SI for the signals (SI_2) quantifies the phase uniformity of the signal. A low SI_2 demonstrates that the signal is periodic and that all phases are equally represented. The numbers below each subplot denote the average cycle duration (mean and standard deviation). B) The inter-seizure interval. We set the lead seizure cut-off at 1 day.

Patient 8

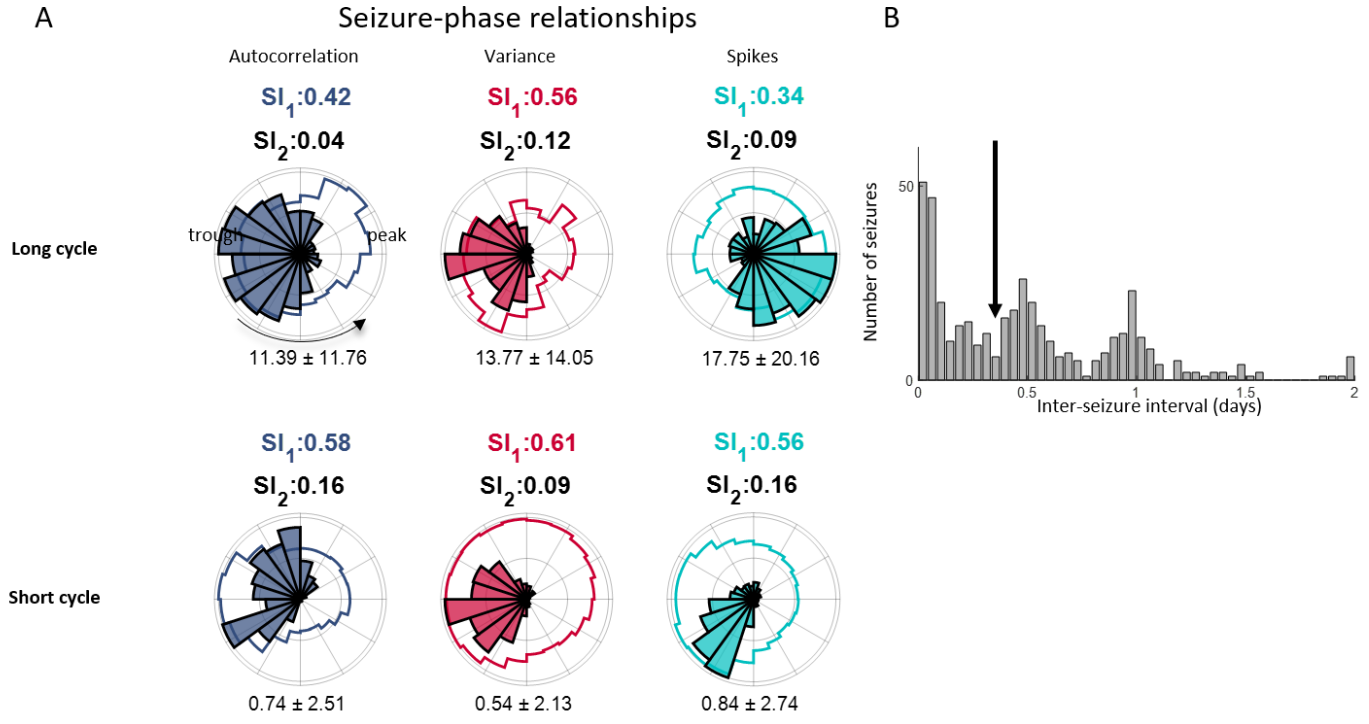

Supplementary Figure 12: Results summary for Patient 8. A) The Synchronization Indices (SI_1) for the autocorrelation (gray-blue), variance (red) and spikes (cyan) quantify the synchrony between seizures and the underlying signals. A high SI_1 corresponds to good alignment between seizures and the phase of the signal. The SI for the signals (SI_2) quantifies the phase uniformity of the signal. A low SI_2 demonstrates that the signal is periodic and that all phases are equally represented. The numbers below each subplot denote the average cycle duration (mean and standard deviation). B) The inter-seizure interval. This patient had multiple peaks separated by approximately 12 hours. We set the lead seizure cut-off to 0.3 days (7 hours).

Patient 9

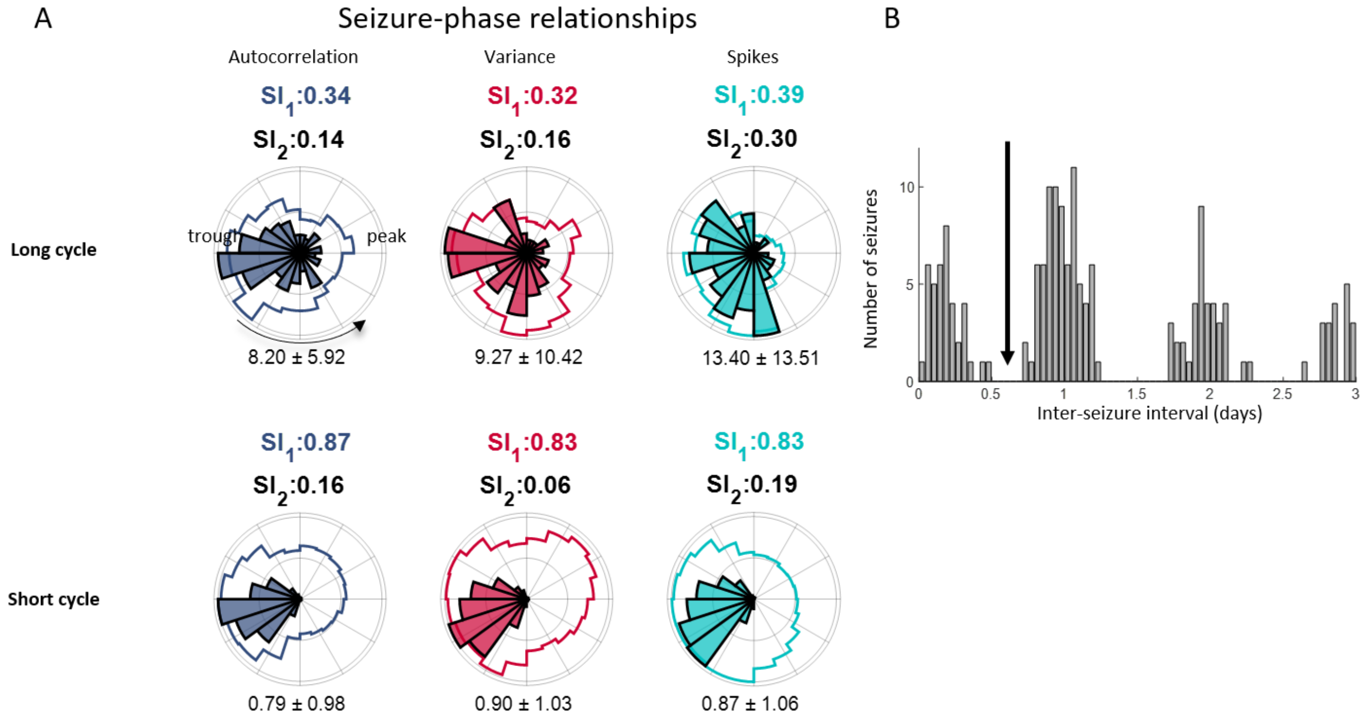

Supplementary Figure 13: Results summary for Patient 9. A) The Synchronization Indices (SI_1) for the autocorrelation (gray-blue), variance (red) and spikes (cyan) quantify the synchrony between seizures and the underlying signals. A high SI_1 corresponds to good alignment between seizures and the phase of the signal. The SI for the signals (SI_2) quantifies the phase uniformity of the signal. A low SI_2 demonstrates that the signal is periodic and that all phases are equally represented. The numbers below each subplot denote the average cycle duration (mean and standard deviation). B) The inter-seizure interval. This patient had multiple peaks separated by approximately 1 day. We set the lead seizure cut-off to 0.7 days (19 hours).

Patient 10

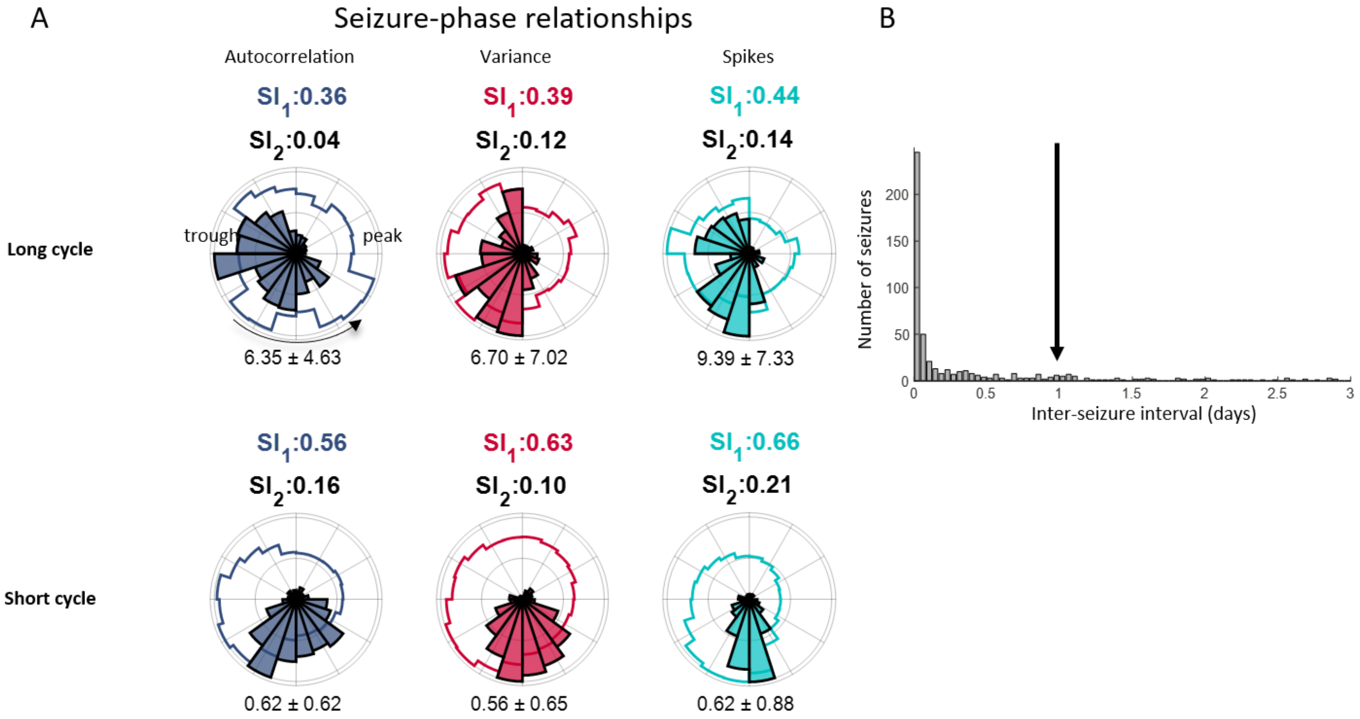

Supplementary Figure 14: Results summary for Patient 10. A) The Synchronization Indices (SI_1) for the autocorrelation (gray-blue), variance (red) and spikes (cyan) quantify the synchrony between seizures and the underlying signals. A high SI_1 corresponds to good alignment between seizures and the phase of the signal. The SI for the signals (SI_2) quantifies the phase uniformity of the signal. A low SI_2 demonstrates that the signal is periodic and that all phases are equally represented. The numbers below each subplot denote the average cycle duration (mean and standard deviation). B) The inter-seizure interval. We set the lead seizure cut-off to 1 day.

Patient 11

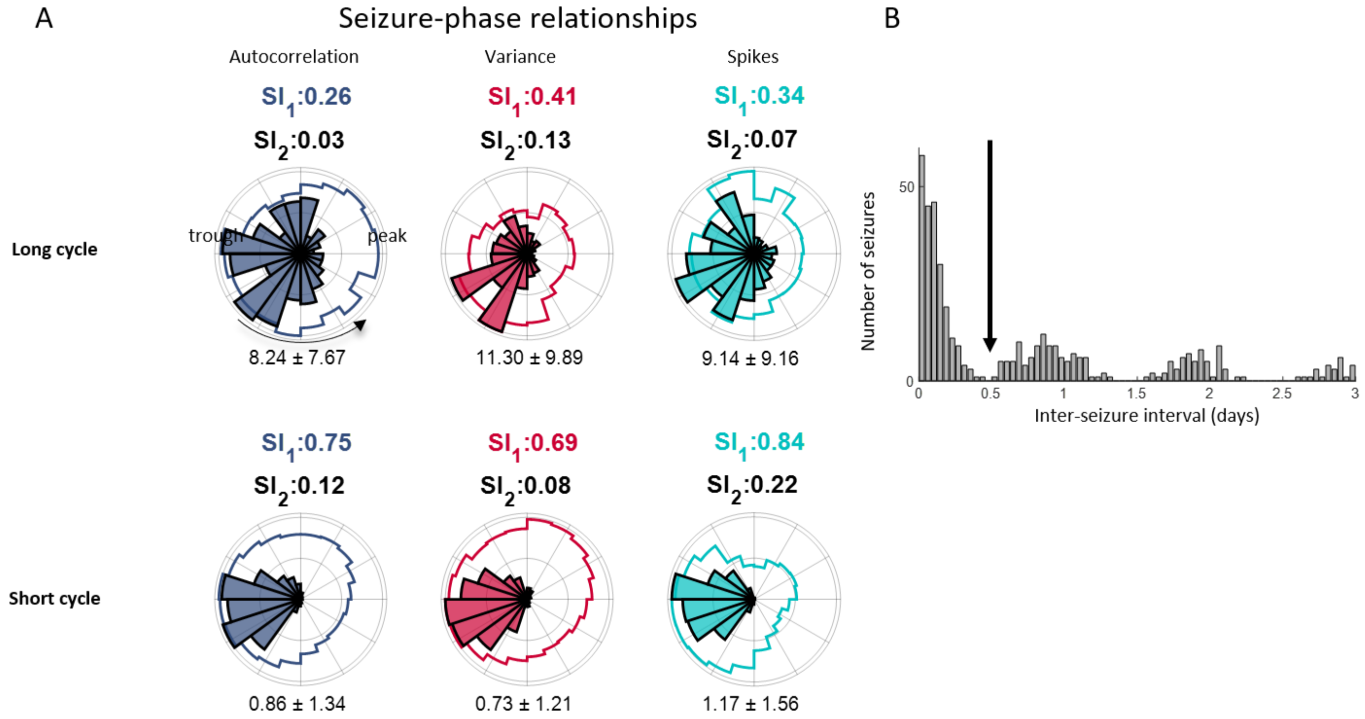

Supplementary Figure 15: Results summary for Patient 11. A) The Synchronization Indices (SI_1) for the autocorrelation (gray-blue), variance (red) and spikes (cyan) quantify the synchrony between seizures and the underlying signals. A high SI_1 corresponds to good alignment between seizures and the phase of the signal. The SI for the signals (SI_2) quantifies the phase uniformity of the signal. A low SI_2 demonstrates that the signal is periodic and that all phases are equally represented. The numbers below each subplot denote the average cycle duration (mean and standard deviation). B) The inter-seizure interval. This patient had multiple peaks separated by approximately 1 day. We set the lead seizure cut-off to 0.5 days (12 hours).

Patient 12

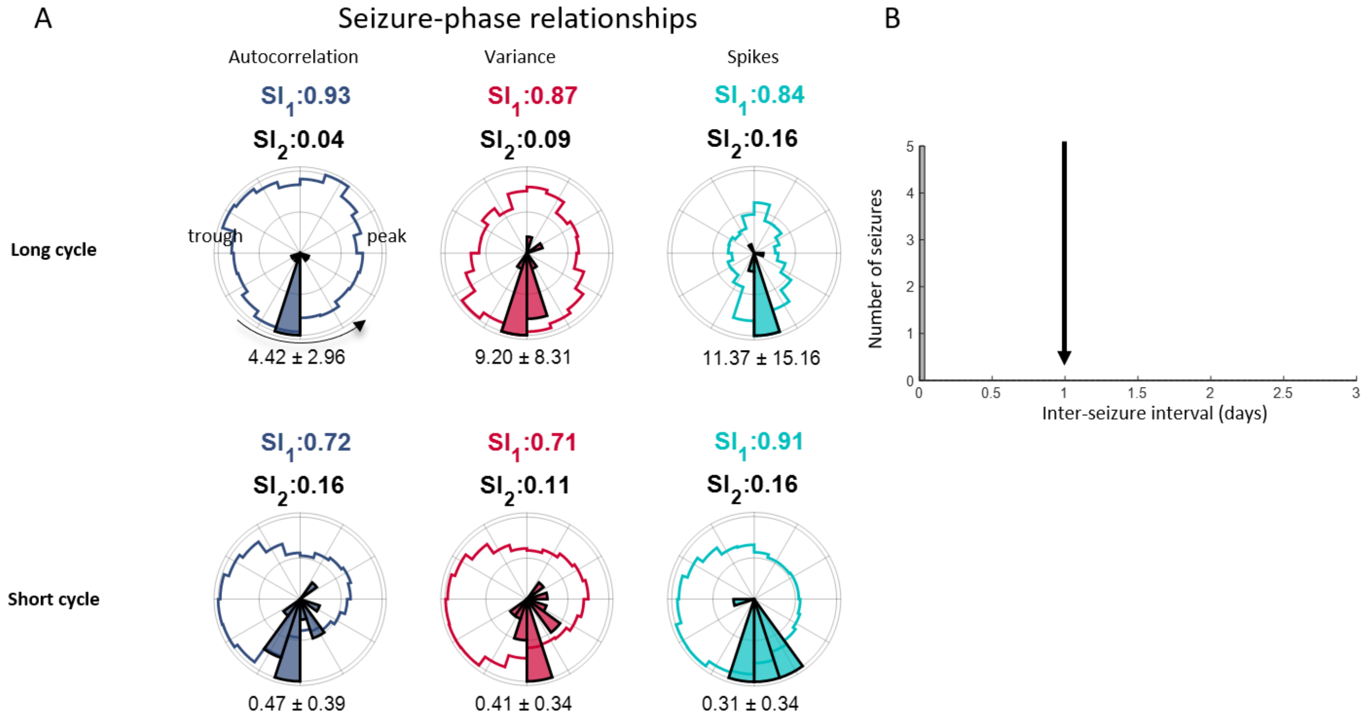

Supplementary Figure 16: Results summary for Patient 12. A) The Synchronization Indices (SI_1) for the autocorrelation (gray-blue), variance (red) and spikes (cyan) quantify the synchrony between seizures and the underlying signals. A high SI_1 corresponds to good alignment between seizures and the phase of the signal. The SI for the signals (SI_2) quantifies the phase uniformity of the signal. A low SI_2 demonstrates that the signal is periodic and that all phases are equally represented. The numbers below each subplot denote the average cycle duration (mean and standard deviation). B) The inter-seizure interval showed that most seizures occurred within 1 hour of a previous seizure. We set the lead seizure cut-off at 1 day.

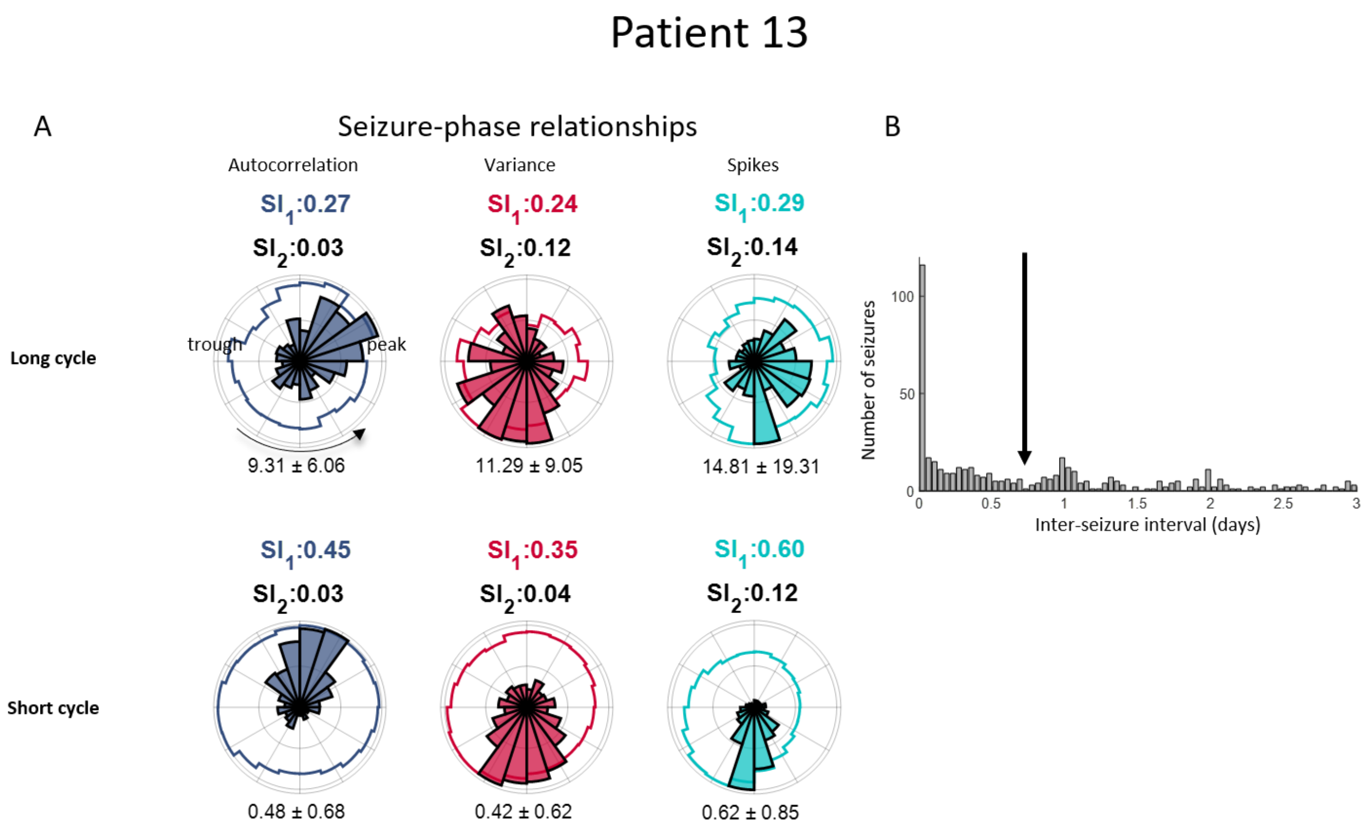

Supplementary Figure 17: Results summary for Patient 13. A) The Synchronization Indices (SI_1) for the autocorrelation (gray-blue), variance (red) and spikes (cyan) quantify the synchrony between seizures and the underlying signals. A high SI_1 corresponds to good alignment between seizures and the phase of the signal. The SI for the signals (SI_2) quantifies the phase uniformity of the signal. A low SI_2 demonstrates that the signal is periodic and that all phases are equally represented. The numbers below each subplot denote the average cycle duration (mean and standard deviation). B) The inter-seizure interval. This patient had peaks at approximately 1 and 2 days. We set the cut-off at 0.7 days (17 hours).

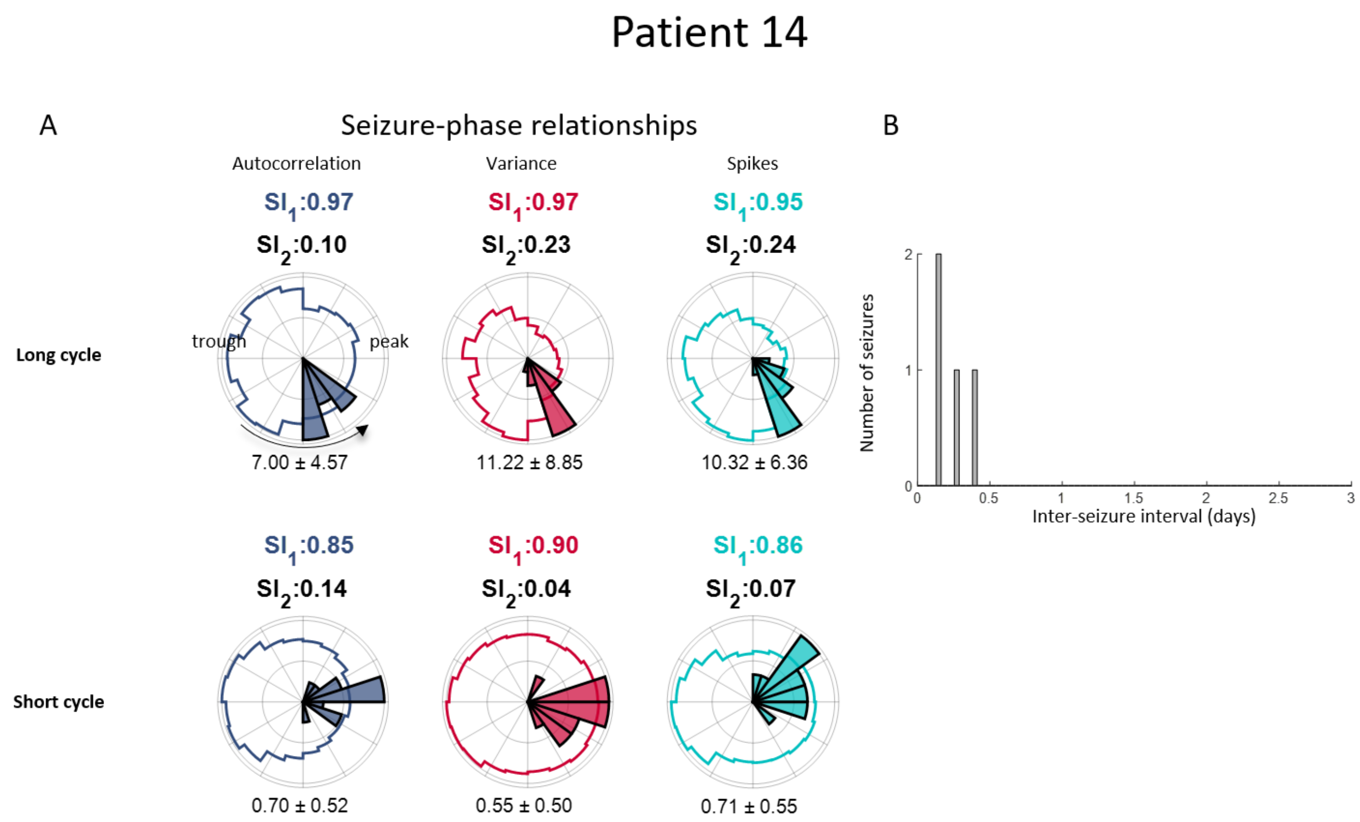

Supplementary Figure 18: Results summary for Patient 14. A) The Synchronization Indices (SI_1) for the autocorrelation (gray-blue), variance (red) and spikes (cyan) quantify the synchrony between seizures and the underlying signals. A high SI_1 corresponds to good alignment between seizures and the phase of the signal. The SI for the signals (SI_2) quantifies the phase uniformity of the signal. A low SI_2 demonstrates that the signal is periodic and that all phases are equally represented. The numbers below each subplot denote the average cycle duration (mean and standard deviation). B) The inter-seizure interval. This patient had too few seizures to meet the seizure clustering criteria.

Patient 15

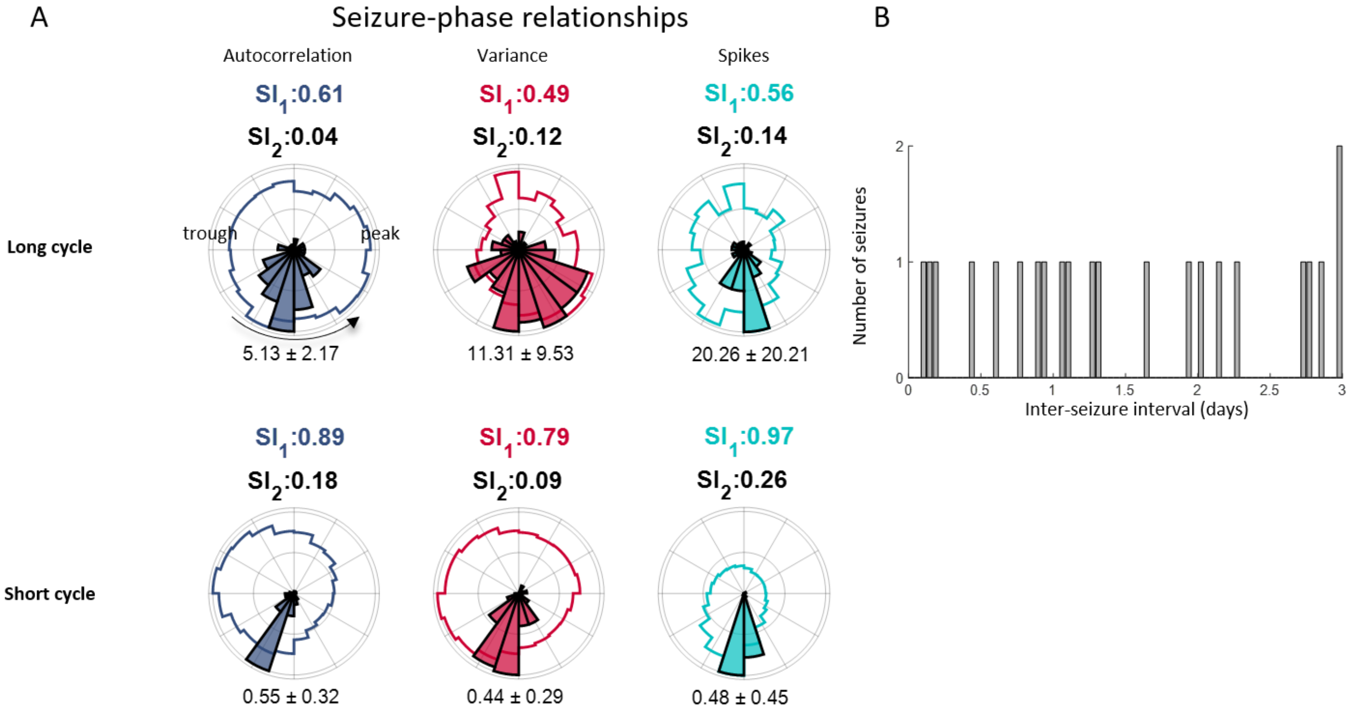

Supplementary Figure 19: Results summary for Patient 15. A) The Synchronization Indices (SI_1) for the autocorrelation (gray-blue), variance (red) and spikes (cyan) quantify the synchrony between seizures and the underlying signals. A high SI_1 corresponds to good alignment between seizures and the phase of the signal. The SI for the signals (SI_2) quantifies the phase uniformity of the signal. A low SI_2 demonstrates that the signal is periodic and that all phases are equally represented. The numbers below each subplot denote the average cycle duration (mean and standard deviation). B) The inter-seizure interval. This patient had too few seizures to meet the seizure clustering criteria.

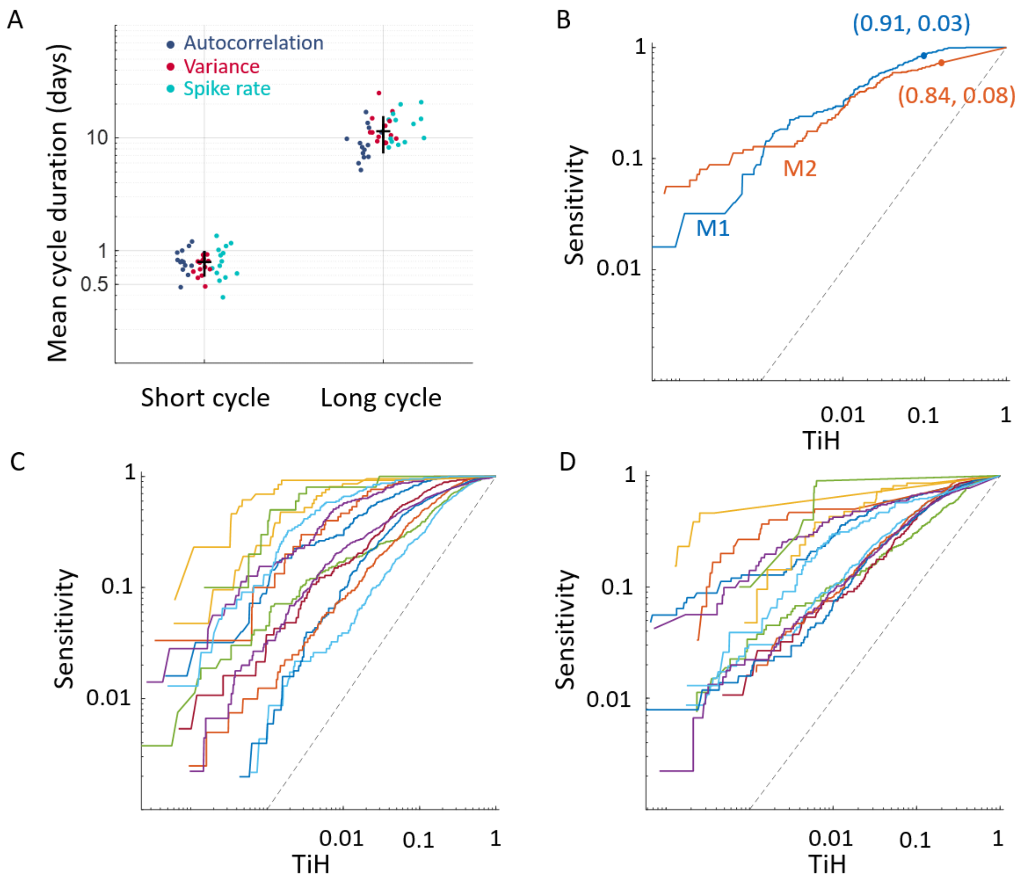

Supplementary Figure 20: Cycle durations and forecasting performance. A) The average short and long cycle duration is shown across all patients (black cross). Each colored dot represents the cycle duration for autocorrelation (blue), variance (red) and spike rates (cyan). B) The Receiver Operating Characteristic (ROC) curve for methods M1 and M2 in patient 1. Also highlighted are the optimal operating points as determined by our optimization algorithm. C) The ROC curves for all patients using method M1. D) The ROC curves for all patients using method M2. The gray dashed line represents the chance level. Note that the axes are presented in log scale to visually enhance the range of small values.

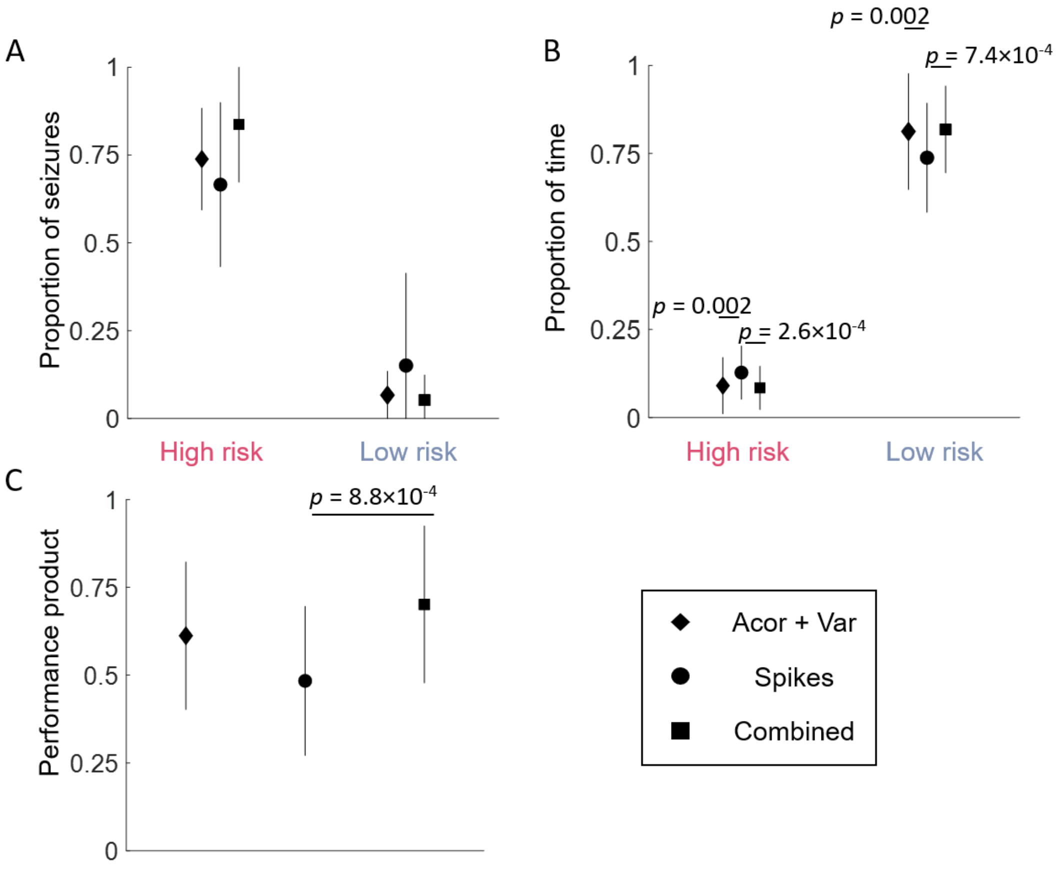

Supplementary Figure 21: Forecasting performance was compared for critical slowing (autocorrelation and variance), spikes and the combination of the three measures. A) there were no significant differences in the number of seizures correctly classified as high risk, or the number of seizures occurring during low risk. B) There was a significantly higher proportion of time spent in the high risk state, and significantly lower amount of time in the low risk state using the spike rate model than the other two models. C) A performance product was used to compare the three different cases. There was a significant effect of method on performance ($F_{2,12} = 8.9$; $p = 0.0013$), and the combined approach performed significantly better than using spike rates alone ($p = 9\times10^{-4}$). Symbols represent means across patients and bars indicate ±one standard deviation (N = 14). Statistical comparisons were computed using a balanced 2-way ANOVA corrected with a Tukey-Kramer multiple comparisons test.

Supplementary Table 1: Summary of forecasting results for Method M1 and M2. Where available, these results are compared to the original trial [1], which included a training and advisory phase.

Patient	Method	Seizures in Low	Seizures in High	Time in Low	Time in High
1	M1	3	91	95	3
	M2	9	83	91	8
	OT† training		75	27	33
	OT advisory		77	7	27
2	M1	9	78	93	3
	M2	9	88	100	2×10^{-3}
	OT training		75	58	21
	OT advisory		100	56	31
4	M1	9	86	99	0.6
	M2	14	73	100	2×10^{-3}
6	M1	0	94	99	0.6
	M2	27	66	95	3
7	M1	13	69	79	10
	M2	22	64	66	23
8	M1	15	61	72	13
	M2	12	72	83	11
	OT training		63		40
	OT advisory		62		28
9	M1	2	88	69	15
	M2	7	85	81	16
	OT training		59	19	36
	OT advisory		17	48	11
10	M1	7	80	77	11
	M2	11	79	66	24
	OT training		75		31
	OT advisory		51		17
11	M1	6	76	76	12
	M2	6	86	81	16
	OT training		65	20	30
	OT advisory		39	26	15
12	M1	0	71	100	2×10^{-4}
	M2	15	85	100	2×10^{-4}
13	M1	10	70	67	14
	M2	11	69	79	10
	OT training		62		35
	OT advisory		50		28
14	M1	0	73	100	5×10^{-4}
	M2	8	67	100	2×10^{-3}
15	M1	0	97	99	0.8
	M2	6	87	89	7
	OT training		100		18
	OT advisory		71		41
Total⸸	M1	5 ± 7	84 ± 16	82 ± 12	8 ± 6
	M2	13 ± 6	77 ± 8	87 ± 12	9 ± 8
	OT training		72 ± 13	31 ± 18	31 ± 8
	OT advisory		58 ± 25	34 ± 22	25 ± 10

All values in the table represent percentages rounded to the closest integer.

† OT – original trial. Where available, sensitivity (seizures in high), time in high and time in low values from the original trial in the training and advisory phases are shown [1].

⸸ Values represent mean ± 1 standard deviation.

Supplementary Table 2: Comparison of NeuroVista data studies – Sensitivity (S)/Time in high (TiH)

Patient	Original [1]		Deep CNN [2]		CW [3]		Logistic [3]		CW + Logistic [3]		Kaggle [4]		CW + Kaggle [4]		Critical slowing	
	S	TiH	S	TiH	S	TiH	S	TiH	S	TiH	S	TiH	S	TiH	S	TiH
1	77	27	65	21	34	27	54	27	61	27					68	7
2	100	31	74	11											75	0.7
3	45	29	71	53	36	29	53	29	55	29	66	29	60	29		
4															64	0.02
6					52		61		65						72	2
7															76	21
8	62	28	77	32	58	28	71	28	76	28					67	14
9	17	11	83	43	28	11	29	11	45	11	39	11	52	11	75	10
10	51	17	68	32	36	17	38	17	52	17	48	17	53	17	69	13
11	39	15	78	18	43	15	57	15	58	15					83	17
12															54	2×10^{-5}
13	50	28	70	21	61	28	78	28	76	28					64	14
14	100	3	42	2											75	6×10^{-4}
15	71	21	59	37	71	21	51	21	60	21					88	2
Average	61 (52)	21 (22)	69 (71)	27 (32)	(47)	(21)	(57)	(21)	(61)	(21)					72 (71)	8 (9)

- Best method in green
- Averages in parentheses ignore patients 2 and 14

Supplementary References

1. Cook, M.J., T.J. O'Brien, S.F. Berkovic, et al., *Prediction of seizure likelihood with a long-term, implanted seizure advisory system in patients with drug-resistant epilepsy: A first-in-man study.* The Lancet Neurology. 12, p. 563-571, 2013
2. Kiral-Kornek, I., S. Roy, E. Nurse, et al., *Epileptic Seizure Prediction Using Big Data and Deep Learning: Toward a Mobile System.* EBioMedicine. 27, p. 103-111, 2018
3. Karoly, P.J., H. Ung, D.B. Grayden, et al., *The circadian profile of epilepsy improves seizure forecasting.* Brain. 140, p. 2169-2182, 2017
4. Kuhlmann, L., P. Karoly, D.R. Freestone, et al., *Epilepsyecosystem.org: Crowd-sourcing reproducible seizure prediction with long-term human intracranial EEG.* Brain. 141, p. 2619-2630, 2018
