# Supplementary material for: Critical slowing down as a biomarker for seizure susceptibility
Source: Nat Commun. 2020 May 1;11:2172. doi: 10.1038/s41467-020-15908-3 (PMC7195436; doi:10.1038/s41467-020-15908-3)
Supplement: Supplementary file 3 — Reporting summary [file 41467_2020_15908_MOESM3_ESM.pdf]

nature research

Corresponding author(s): Matias Ismael Maturana

Last updated by author(s): 10 Feb 2020

# Reporting Summary

Nature Research wishes to improve the reproducibility of the work that we publish. This form provides structure for consistency and transparency in reporting. For further information on Nature Research policies, see Authors & Referees and the Editorial Policy Checklist .

## Statistics

For all statistical analyses, confirm that the following items are present in the figure legend, table legend, main text, or Methods section.

| n/a | Confirmed | |
|---|---|---|
| ☐ | ☒ | The exact sample size (*n*) for each experimental group/condition, given as a discrete number and unit of measurement |
| ☐ | ☒ | A statement on whether measurements were taken from distinct samples or whether the same sample was measured repeatedly |
| ☐ | ☒ | The statistical test(s) used AND whether they are one- or two-sided<br>*Only common tests should be described solely by name; describe more complex techniques in the Methods section.* |
| ☒ | ☐ | A description of all covariates tested |
| ☐ | ☒ | A description of any assumptions or corrections, such as tests of normality and adjustment for multiple comparisons |
| ☐ | ☒ | A full description of the statistical parameters including central tendency (e.g. means) or other basic estimates (e.g. regression coefficient) AND variation (e.g. standard deviation) or associated estimates of uncertainty (e.g. confidence intervals) |
| ☐ | ☒ | For null hypothesis testing, the test statistic (e.g. *F*, *t*, *r*) with confidence intervals, effect sizes, degrees of freedom and *P* value noted<br>*Give P values as exact values whenever suitable.* |
| ☒ | ☐ | For Bayesian analysis, information on the choice of priors and Markov chain Monte Carlo settings |
| ☒ | ☐ | For hierarchical and complex designs, identification of the appropriate level for tests and full reporting of outcomes |
| ☒ | ☐ | Estimates of effect sizes (e.g. Cohen's *d*, Pearson's *r*), indicating how they were calculated |

*Our web collection on statistics for biologists contains articles on many of the points above.*

## Software and code

Policy information about availability of computer code

| | |
|---|---|
| Data collection | Data was collected as part of a separate clinical trial (Cook et.al., 2013). Data was store and accessed from iEEG.org - an online platform for data sharing and analysis. |
| Data analysis | Data was analyzed using Matlab version 2017b using inbuilt functions and custom code.<br>Code used in this study has been made available on github. Please see:<br>https://github.com/matiasim/Critical_Slowing_Epilepsy |

For manuscripts utilizing custom algorithms or software that are central to the research but not yet described in published literature, software must be made available to editors/reviewers. We strongly encourage code deposition in a community repository (e.g. GitHub). See the Nature Research guidelines for submitting code & software for further information.

## Data

Policy information about availability of data

All manuscripts must include a data availability statement. This statement should provide the following information, where applicable:

- Accession codes, unique identifiers, or web links for publicly available datasets
- A list of figures that have associated raw data
- A description of any restrictions on data availability

Due to the nature of the data (clinical trial data), not all is available to the public. However, a big portion of the data has been recently made publicly available on https://www.epilepsyecosystem.org/. The remaining data can be made available under a collaborative agreement upon reasonable request.

# Field-specific reporting

Please select the one below that is the best fit for your research. If you are not sure, read the appropriate sections before making your selection.

☒ Life sciences ☐ Behavioural & social sciences ☐ Ecological, evolutionary & environmental sciences

For a reference copy of the document with all sections, see nature.com/documents/nr-reporting-summary-flat.pdf

# Life sciences study design

All studies must disclose on these points even when the disclosure is negative.

| | |
|---|---|
| Sample size | Data was taken from a clinical trial involving 15 patients implanted with an ECoG device. The current study was retrospective. |
| Data exclusions | Data from one patient (patient 3) was excluded from the analysis. The data from this patient had large portions of missing data making an analysis of long term rhythms impossible. No other data was excluded. |
| Replication | All analysis can be reproduced using the software, analysis methods, code and data mentioned in the paper. Experimental data used in the study cannot be reproduced since it was retrospective data collected from a previous clinical trial. |
| Randomization | Not relevant to the study since it was retrospective on data collected from a previous clinical trial. The trial selection criteria has been extensively documented (Cook et. al. 2013). |
| Blinding | Not relevant to the study since it was retrospective on data collected from a previous clinical trial. The trial selection criteria has been extensively documented (Cook et. al. 2013). |

# Reporting for specific materials, systems and methods

We require information from authors about some types of materials, experimental systems and methods used in many studies. Here, indicate whether each material, system or method listed is relevant to your study. If you are not sure if a list item applies to your research, read the appropriate section before selecting a response.

## Materials & experimental systems

| n/a | Involved in the study | |
|---|---|---|
| ☒ | ☐ | Antibodies |
| ☒ | ☐ | Eukaryotic cell lines |
| ☒ | ☐ | Palaeontology |
| ☒ | ☐ | Animals and other organisms |
| ☒ | ☐ | Human research participants |
| ☐ | ☒ | Clinical data |

## Methods

| n/a | Involved in the study | |
|---|---|---|
| ☒ | ☐ | ChIP-seq |
| ☒ | ☐ | Flow cytometry |
| ☒ | ☐ | MRI-based neuroimaging |

## Clinical data

Policy information about clinical studies

All manuscripts should comply with the ICMJEguidelines for publication of clinical research and a completedCONSORT checklist must be included with all submissions.

| | |
|---|---|
| Clinical trial registration | NIH clinical trial number: NCT01043406. St Vincent's Hospital Clinical human research ethics approval number: HREC-D 147/09 |
| Study protocol | Cook MJ, O'Brien TJ, Berkovic SF, Murphy M, Morokoff A, Fabinyi G, D'Souza W, Yerra R, Archer J, Litewka L, Hosking S. Prediction of seizure likelihood with a long-term, implanted seizure advisory system in patients with drug-resistant epilepsy: a first-in-man study. The Lancet Neurology. 2013 Jun 1;12(6):563-71. |
| Data collection | Data was collected from three hospitals in the state of Victoria, Australia. Data was collected during the period between 2009 and 2012. |
| Outcomes | Outcome measures from the clinical trial have been extensively described in Cook et.al. 2013. Furthermore, a STROBE checklist was provided in the original study. Since the original study was based on safety, primary outcome measures were the number of device related adverse effects after 4 months. secondary endpoints were algorithm performance at the end of the data collection phase, clinical effectiveness (measures of anxiety, depression, seizure severity, and quality of life) 4 months after iniation of the advisory phase, and longer-term adverse events |
